# Thermal Comfort of Nelore Cattle (*Bos indicus*) Managed in Silvopastoral and Traditional Systems Associated with Rumination in a Humid Tropical Environment in the Eastern Amazon, Brazil

**DOI:** 10.3390/vetsci11060236

**Published:** 2024-05-23

**Authors:** Welligton Conceição da Silva, Jamile Andréa Rodrigues da Silva, Lucieta Guerreiro Martorano, Éder Bruno Rebelo da Silva, Katarina Cardoso de Carvalho, Carlos Eduardo Lima Sousa, Kedson Alessandri Lobo Neves, Raimundo Nonato Colares Camargo Júnior, Tatiane Silva Belo, Ana Gizela Souza de Santos, Cláudio Vieira de Araújo, Leonel António Joaquim, Thomaz Cyro Guimarães de Carvalho Rodrigues, José de Brito Lourenço-Júnior

**Affiliations:** 1 Postgraduate Program in Animal Science (PPGCAN), Institute of Veterinary Medicine, Federal University of Para (UFPA), Castanhal 68740-970, PA, Brazil; eder.b.rebelo@gmail.com (É.B.R.d.S.); camargojunior@gmail.com (R.N.C.C.J.); jleokim.a@hotmail.com (L.A.J.); thomazguimaraes@yahoo.com.br (T.C.G.d.C.R.); joselourencojr@yahoo.com.br (J.d.B.L.-J.); 2 Institute of Animal Health and Production, Federal Rural University of the Amazon (UFRA), Belem 66077-580, PA, Brazil; jamileandrea@yahoo.com.br; 3 Embrapa Eastern Amazon, Santarem 68010-180, PA, Brazil; lucieta.martorano@embrapa.br; 4 Department of Veterinary Medicine, University Center of the Amazon (UNAMA), Santarem 68010-200, PA, Brazil; katarinacc4@gmail.com (K.C.d.C.); cadu34.medvet@gmail.com (C.E.L.S.); tatianebelovet@gmail.com (T.S.B.); gizelamedvet@gmail.com (A.G.S.d.S.); 5 Institute of Animal Science, Federal University of Western Pará (UFOPA), Santarem 68040-255, PA, Brazil; kedson_neves@hotmail.com; 6 Department of Agricultural and Environmental Sciences, Federal University of Mato Grosso (UFMT), Sinop 78550-728, MT, Brazil; cvaufmt@gmail.com

**Keywords:** thermal stress, respiratory rate, climate, cattle, cattle welfare

## Abstract

**Simple Summary:**

The objective of this study was to evaluate the thermal comfort of Nelore cattle (*Bos indicus*) managed in silvopastoral and traditional systems associated with rumination behavior in a humid tropical environment in the Eastern Amazon, Brazil. The thermoregulatory responses of 20 uncastrated male Nelore cattle in silvopastoral and traditional systems from June to July 2023 were evaluated. Physiological variables were measured, including respiratory rate (RR), rectal temperature (RT) and body surface temperature (BST). The RR was higher in the traditional system and RT showed significant variations over the collection periods. The black globe temperature and humidity index (BGHI) indicated mild to moderate stress. The silvopastoral system showed advantages in RR, RT and in rumination behavior. The results suggest that air temperature (AT) significantly influenced RR and thermal comfort in both systems, and that the SP system offers more thermal comfort advantages compared to the TS system.

**Abstract:**

The objective of this study was to evaluate the thermal comfort of Nelore cattle (*Bos indicus*) managed in silvopastoral and traditional systems associated with rumination behavior in a humid tropical environment in the Eastern Amazon, Brazil. The study was carried out on a rural property in Mojuí dos Campos, Pará, Brazil, during the transition period of the year, from June to July 2023. Over these two months, six consecutive data collection days were held. We selected 20 clinically healthy non-castrated male Nelore cattle, aged between 18 and 20 months, with an average weight of 250 kg and body condition score of 3.5 (1–5). These animals were randomly divided into two groups: traditional system (TS) and silvopastoral system (SS). The physiological variables evaluated included RR, RT and BST. The variables were analyzed using the linear mixed model. For agrometeorological variables, higher values were observed between 10:00 a.m. (33 °C) and 6:00 p.m. (30 °C), with the highest temperature observed at 4:00 p.m. (40 °C). The RR showed interactions (*p* = 0.0214) between systems and times; in general, higher RR were obtained in the Traditional. The animals’ RT showed no significant difference (*p* < 0.05) between the production systems, but there was a statistically significant difference in relation to the time of collection (*p* < 0.0001). In the BGHI, it was possible to observe that there was mild stress in the period from 22:00 at night to 6:00 in the morning and moderate stress in the period of greatest increase in temperature, from 10:00 in the morning to 18:00 at night. BST showed no statistical difference between the regions studied or between the SP (35.6 °C) and TS (36.25 °C) systems. RT in the TS showed a positive correlation with AT (r = 0.31507; *p* = 0.0477). RT in the SP showed a positive correlation with THI (r = 35583; *p* = 0.0242). On the other hand, RT in the SP (r = 0.42873; *p* = 0.0058) and ST (r = 0.51015; *p* = 0.0008) showed a positive correlation with BGHI. RR in the TS showed a positive correlation with BGHI (r = 0.44908; *p* = 0.0037). The greatest amounts of rumination were carried out by animals in the SP system, generally ruminating lying down (*p* < 0.05). With regard to rumination behavior in the morning and afternoon, there were higher numbers of WS and LD in the TS (*p* > 0.05). Most of the time, the cattle were LD during the morning and afternoon shifts, and at night and dawn they were WS in the TS. Therefore, the SP offers more thermal comfort advantages compared to the TS system.

## 1. Introduction

Animal welfare is defined as the mental and physical state in relation to the environment in which they live and die [1,2]. Therefore, a good degree of animal welfare demonstrates that an individual is healthy, safe, comfortable, well nourished and free to express natural behaviors of the species without suffering from harmful psychological states such as frustration, pain and stress [3,4,5].

Animals have the ability to control their body temperature when exposed to high temperature variations, with thermoregulation being the mechanism responsible for homeostasis, dissipating excess heat accumulated in the body through perspiration and peripheral circulation, resulting in increased breathing, wheezing and decreased rates of food intake to retain metabolic heat [2,3,4,5,6,7,8,9,10,11].

Therefore, one of the most common causes capable of reducing animal welfare is thermal conditions, i.e., very low or very high temperatures, due to anatomical reasons or the environment in which the animals are raised [12,13,14,15,16,17,18]. The animal organism tends to prioritize homeostasis; however, when subjected to agents that trigger stress, animals respond through a combination of physiological, biochemical and behavioral reactions [19,20,21].

Thermal stress causes serious negative effects on the welfare of cattle, which can lead to major economic and large-scale production losses [22]. Furthermore, sweating, increased RR, water intake, vasodilation, reduced productivity and decreased milk production may occur, and in high degrees of stress, an increased mortality rate may be seen [23,24,25,26,27].

In this context, infrared thermography appears to be a non-invasive method capable of capturing the surface temperature of animals and helping to identify the increase in temperature [2,28,29,30,31,32,33,34,35]. Infrared thermography has proven to be a non-invasive and accurate technique capable of identifying changes in surface temperature, as well as preventing animal stress and promoting animal welfare [17,18,30,31,32]. This technique can be used for production, companion and laboratory animals [31]. As for physiological mechanisms, the diameter of the blood vessels located near the body surface is considered one of the main mechanisms responsible for heat loss or gain, i.e., skin vasodilation provides greater heat exchange with the environment [32,33].

Furthermore, different mathematical indices can be used to measure the degree of stress in cattle, such as the Temperature and Humidity Index (THI), the Black Globe and Humidity Index (BGHI) and the Benezra index [36,37,38,39].

The evaluation of several mathematical indices, including the THI, BGHI and the Benezra index, makes it possible to understand and monitor the thermoregulatory responses of cattle. These indices provide quantitative measures to assess the degree of stress experienced by animals in response to environmental conditions [36].

The THI, for example, considers the combined effects of temperature and humidity, offering a comprehensive indicator of heat stress. Similarly, the BGHI takes into account the temperature of the globe, which incorporates radiant heat, adding another layer of complexity to the assessment of environmental stress in livestock [39].

Calculating these indices provides information on the thermoregulatory capacity faced by cattle in different environments. Understanding how temperature, humidity and other factors interact allows the development of specific strategies to mitigate heat stress, improving animal welfare and optimizing productivity [36].

The reduction in heat stress in grazing animals involves applying various strategies to mitigate the adverse effects of high temperatures. It is essential to provide ample shade, as this allows animals to escape direct sunlight and reduces the ambient temperature. Adequate access to fresh, clean water is important to prevent dehydration, and water sources should be strategically placed throughout the pasture. In addition, promoting good air circulation, maintaining adequate spacing between animals and using natural windbreaks can help dissipate heat.

Ruminating behavior is characterized by chewing, regurgitation and remastication, and is also a process by which animals adapt to heat stress conditions. During heat stress, animals tend to ruminate more, since the production of saliva acts as an alternative means to dissipate the heat absorbed by the animal, avoiding hyperthermia [17].

In hot environments, extreme heat can lead to heat stress and reduce the frequency of rumination behavior, due to the overload of the animal’s thermoregulation mechanism, causing it to use energy-intensive resources to try to dissipate the heat, consequently reducing the digestibility, performance and health of ruminants [30,31,32].

However, there are limitations to managing heat stress in pastures, including the inability to control the ambient temperature. In addition, the availability of shade and water can be compromised in extensive grazing systems. Monitoring weather forecasts and adjusting grazing schedules can help minimize heat stress, but the challenges of unpredictable environmental conditions remain a constant consideration when managing animals on pasture.

This study is justified by the need to understand the impact of silvopastoral and traditional systems on the physiological variables of Nelore cattle in the challenging Amazonian environment. The information acquired has the potential to guide the development of more efficient management practices, promoting animal welfare and productivity under the specific climatic conditions of the region. It should be noted that the traditional system, with animals exposed to the sun, is widely adopted in the Amazon, making this study fundamental for revealing how animals in this region behave under different shading conditions, providing information that will help cattle breeders.

In this scenario, evaluating the behavior of cattle, especially the act of ruminating while lying down or standing up, can indicate a zone of comfort or thermal stress, and is essential for deciding on the best place or system in which to raise the animals [17]. For all these reasons, the objective of this study was to evaluate the thermal comfort of Nelore cattle (*Bos indicus*) managed in silvopastoral and traditional systems associated with rumination behavior in a humid tropical environment in the Eastern Amazon, Brazil.

## 2. Materials and Methods

### 2.1. Ethical Aspects

This study was submitted to the Committee for Ethics in the Use of Animals (CEUA) and obtained Approved status, under protocol CEUA-UNAMA 0001-87/2023, in May 2023.

### 2.2. Location

**Figure 1:**
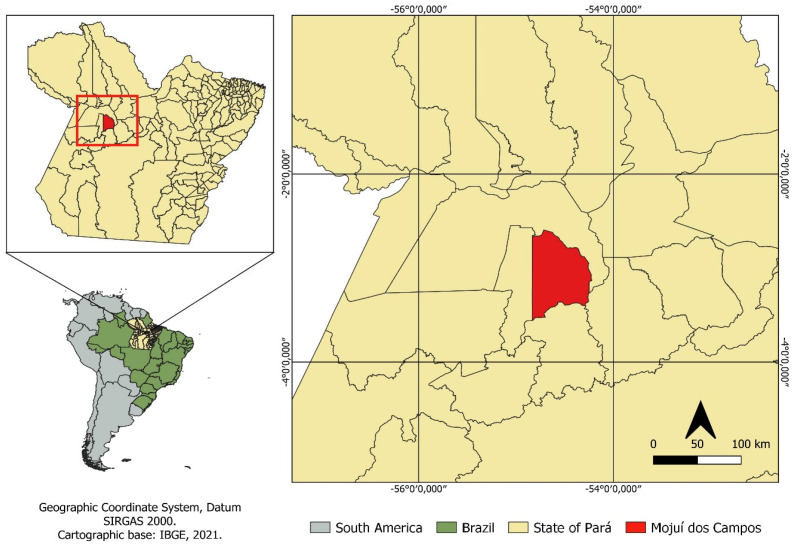
Location map of the study area.

The study was carried out on a rural cattle farm, located in Mojuí dos Campos, Pará, Brazil (Figure 1), in the transition period of the year (rainier to less rainy—June/July).

The climate of the mesoregion is hot-humid (Am4), characterized by total rainfall of less than 60 mm in the least rainy month and with annual rainfall between 1900 and 2100 mm, average annual air temperature classified as 25.6 °C and humidity relative values ranging from 84 to 86% [40]. The rainiest quarter occurs between the months of February and April and the least rainy between the months of August and October [41].

### 2.3. Experimental Animals, Management and Characterization of the Production System

We used 20 clinically healthy, non-castrated male Nelore cattle (*Bos indicus*), with similar coloring, aged between 18 and 20 months, with an average weight of 250 ± 36 kg and body condition score 3.5 (scale from 1 to 5). The animals had white coats, which is common in Nelore cattle raised in the Amazon region.

In this study, only uncastrated males were used due to the common practice in the Amazon region of raising males for fattening, as they are then sent for slaughter. This choice reflects the local reality, where the rearing of males for meat production purposes is predominant. Focusing on the use of non-gelded males allowed for a more specific and representative analysis of the thermal comfort conditions faced by this segment in the region.

The cattle were randomly divided into two groups: traditional system—TS (n = 10) and silvopastoral system—SP (n = 10). The TS group was taken to a paddock without tree shadows, with *Brachiaria brizantha* cv. Marandú pasture (Table 1), access to drinking water and mineral salt ad libitum. The SP group remained in a paddock of the same size, with approximately 20% shade from chestnut trees (*Bertholletia excelsa*), with access to drinking water and mineral salt ad libitum. The traditional and silvopastoral groups were supplemented in the dry season with sorghum and corn silage produced on the property (Table 1), in addition to 0.1% of live weight of concentrated feed (Table 1). The total experimental area was 10.2 ha of *Brachiaria brizantha* cv. Marandú divided into six 1.7 ha paddocks, two per treatment.

The animals’ adaptation period to handling was seven consecutive days, where the animals were taken to the chute to collect data on the physiological variables that were evaluated during the experiment. After the adaptation period, the cattle remained in their respective systems. The production systems used were characterized as follows:

i. Traditional System (TS)—No shade and no access to the bathing area. In this system, the animals are grazed without the presence of trees or other elements that can provide shade, and they do not have access to a bathing area.
ii. Silvopastoral System (SP)—With shade and without access to the bathing area. In this system, the animals are subjected to pasture with the presence of trees and other elements that can provide shade.

### 2.4. Agrometeorological Variables

The agrometeorological variables that were evaluated were air temperature (AT °C), relative air humidity (RH %), wind speed (WS, m s^−1^), dew point temperature (DPT °C), wet bulb temperature (WBT °C), and black globe temperature (BGT °C). They were obtained by using thermal sensors, measured every fifteen minutes, during the experimental period.

### 2.5. Physiological Variables

Physiological data were collected at 6:00 a.m., 12:00 p.m., 6:00 p.m., and 12:00 a.m. during the transitional period of June and July. On collection days, the cattle were led to the management corral and held for 30 min before activities commenced, ensuring minimal interference with physiological variables. The animals were walked to the corral on foot, and confined in brete-type trunks (conventional mechanical trunk plus model from the Coimma^®^ brand - Dracena, São Paulo, Brazil) within sheltered areas to protect them from direct sunlight and rain. Handling occurred in groups of ten, with no fixed order of entry, preventing temporal influences on animal data within the designated time frame. The animals underwent a seven day adaptation period before the experiment so that there would be no interference in the collection of physiological data.

The sampling times of 6:00 a.m., 12:00 p.m., 6:00 p.m. and 12:00 a.m. selected for this study were carefully chosen to cover all the times of greatest and least intensity of heat and humidity throughout the day. This strategic approach is crucial when assessing animal comfort, as it allows significant variations in the thermal conditions faced by the animals to be captured. By considering daily extremes, the results obtained during the collections provide a comprehensive and representative view of the impact of environmental conditions on the thermoregulatory responses of Nelore cattle, contributing to a more complete analysis of their thermal animal welfare.

### 2.6. Respiratory Rate (RR)

The RR was obtained by inspecting and counting the thoracic–abdominal movements, for one minute [42], with the help of a digital stopwatch, in the transition period (June/July) at 6:00 a.m., 12:00 p.m., 6:00 p.m. and midnight. This assessment was carried out by a single, previously trained observer.

### 2.7. Rectal Temperature (RT)

The RT was obtained using a veterinary clinical thermometer (Model-5198.10, Incoterm^®^, São Paulo, Brazil), with a maximum scale of up to 44 °C, inserted transrectally into the animals with the results expressed in degrees Celsius, as described by Dirksen et al. [43]. This evaluation was carried out by a single, previously trained observer.

### 2.8. Infrared Thermography

Infrared thermography was employed to diagnose thermal patterns within the environments of the three production systems. Data collection occurred on 1 July 2023, covering an area of 1.7 hectares per production system. This evaluation was carried out by a single, previously trained observer. Thermographic images were captured using a thermal imaging camera (FLIR T650sc, Wilsonville, OR, USA, 2015) between 12:00 and 15:00, a period characterized by the intense impact of solar radiation on the targets observed in the field research. To accurately reflect Thermal Regulation Index (TRI) fluctuations, images were acquired on the right side of the animals, minimizing interference from ruminal movements. The collected images were stored on a memory card and analyzed using FLIR Tools software (version 6.4), calculating average temperatures for each region with an emissivity set at 0.98. In the systems, thermograms were acquired at an approximate orthogonal distance of 5 m, outside the flight zone of the bull [18].

**Figure 2:**
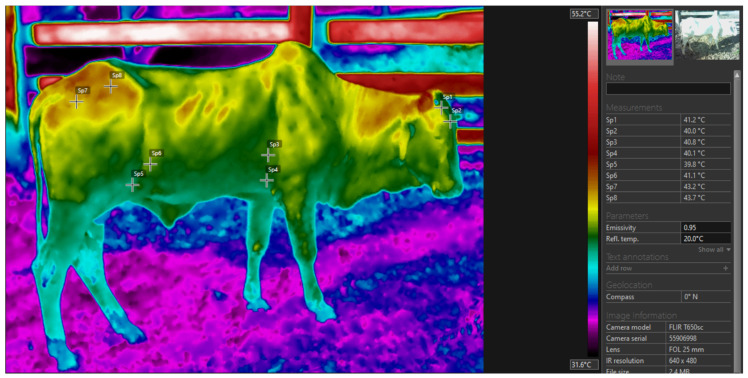
Marking thermal targets using infrared thermography. Description of the anatomical region: SP1 and SP2—head; SP3 and SP4—armpit; SP5 and SP6—flank; and SP7 and SP8—rump.

This camera has high precision, with a fixed 25 mm lens, a temperature range from 40 to 150 °C, thermal sensitivity of 50 mK (>0.05 °C at a room temperature of 30 °C) and a spectral range of coverage from 0.7 to 100 µm, and the imaged targets had a response between 0.7 and 3.0 µm and an optical resolution of 640 × 480 pixels with a maximum emissivity index of 0.95; subsequently, the images were processed in the computer program Flir Tools, 6.3v [44], with the Rainbow HC palette chosen. The images were acquired from four areas, the head region, armpit, flank and rump (Figure 2), according to the description of thermal windows described in Silva et al. [18] and Mota-Rojas et al. [33].

### 2.9. Index to Assess Thermal Comfort

#### 2.9.1. Temperature and Humidity Index (THI)

During the course of the experiment, environmental data were continuously monitored in conjunction with physiological measurements taken throughout the day. Using the recorded environmental parameters, we computed the Temperature and Humidity Index (THI), as proposed by Thom [36], employing the following formula:

THI = DBT + 0.36 × DPT + 41.5

where DBT = dry bulb temperature (°C) and DPT = dew point temperature (°C).

THI = temperature and humidity index, where results of < 72 indicate without stress; 72–78 indicates mild or mild stress; 79–88 indicates moderate stress; and 89–98 indicates severe stress [39].

#### 2.9.2. Black Globe Temperature and Humidity Index (BGHI)

After obtaining these variables, the black globe temperature and humidity index (BGHI) can be calculated, in accordance with what was proposed by Buffington et al. [45], using the following equation:

BGHI = BGT + 0.36 (DPT) + 41.5

where BGHI = black globe temperature and humidity index, BGT = black globe temperature (°C) and DPT = dew point temperature (°C).

BGHI = black globe temperature and humidity index, indicating ≤ 74: thermal comfort situation; 75–78: warning; 79–84: danger; and ≥85: emergency [17].

### 2.10. Behavioral Assessment

The animals were assessed for their ruminating behavior, in which the animal chews, swallows, regurgitates and re-swallows with the food bolus present in the spaces between the cheeks, while standing or lying down. Rumination behavior was observed during the months of June and July. Two of the ten animals assessed were divided up every 5 min, between the hours of 6 a.m. and 6 p.m. [17,46].

The animals were marked on their sides and on the croup with the respective number. Behavior was observed visually and on the spot. A total of six trained observers were used, divided into pairs and replaced every 2 h to avoid fatigue. At the beginning of the experiment, the observers carried out an inter-observer test, i.e., they evaluated and recorded behaviors independently, isolated in the field, for a period of 5 h, thus assessing the accuracy of each evaluator. The inter-observer test was assessed by the Kappa coefficient, calculated using Microsoft Excel 2013 (Microsoft Corp., Redmond, WA, USA), and showed a reliability index of 92.5%, which was adequate, as described by Silva et al. [17].

### 2.11. Data Analysis

The RT and RR parameters collected individually from each animal were considered as independent variables analyzed separately through a linear mixed model with covariance structure in longitudinal data using the following model:

Y = Xb + Za + e

where Y is the vector of animal observations; X is an incidence matrix associated with vector B of the fixed effects of production systems (traditional; silvopastoral) and collections at different times (6:00, 12:00, 18:00 and 00:00 h), such as the interaction between production system and schedules; Z is an incidence matrix associated with the vector a of animal random effects solutions; and “e” is a vector that represents the residuals.

For the residue (“e”), the variance is defined as

$$V(\varepsilon )=I_{\left(np\right)}⊗\Sigma_{O}=R$$

where I is a matrix identity of order “np”, where n is the number of animals and p is the number of measurements taken on each animal for the number of times at which the animals were measured, ⊗ represents the product of Kronecker and Σ_O_ is the structure of the matrix between repeated measurements on the same animal at different times, tested according to the following structures: variance, such as variance component (VC), first order autoregressive (AR(1)), compound symmetry (CS), heterogeneous compound symmetry (CSH) and Toeptiz (Toep).

The choice of the model with the most appropriate residue structure is carried out according to the Akaike information criteria (AIC) [47], Schwars Bayesian (SB), defined as:

AIC = −2log(L) +2p (47) and SB = −2LOG(L) + p log(n)

where p is the number of parameters to be estimated and n is the number of observations in the sample [48]. The structure of the covariance matrix between repeated measures that was most appropriate for analyzing RT and RR was the first-order auto-regressive matrix. The software SAS OnDemand for Academics (version 9.4) [49] was used for statistical analysis by mixed procedure, and in all analyses the significance level was equal to 0.05.

## 3. Results

**Figure 3:**
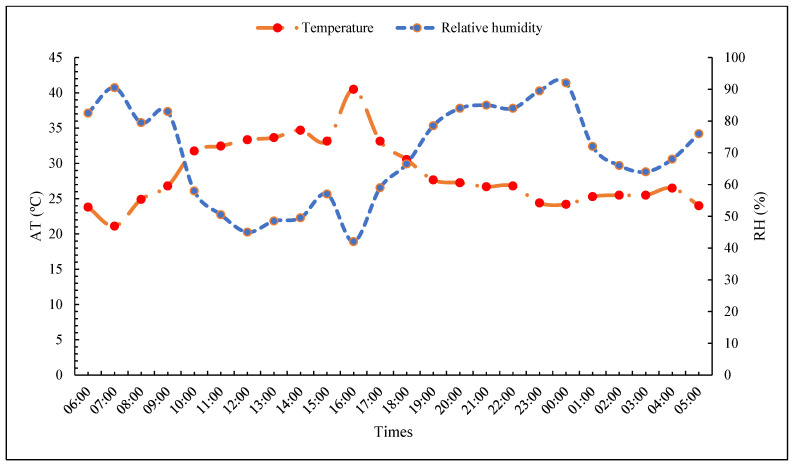
Time and values of the variables relative humidity and air temperature of the climatic variables observed from 6:00 a.m. to 6:00 a.m. during the experimental period, between the months of June and July.

There were changes in the weather variables between the different times of the months, with an oscillation in temperature (Figure 3), with the highest values being observed between 10:00 a.m. (33 °C) and 6:00 p.m. (30 °C), with the highest temperature observed at 16:00 (40 °C). With regard to relative humidity, it was possible to observe a greater rate of fluctuation, with the lowest humidity values observed between 10:00 a.m. (25 °C) and 6:00 p.m. (26 °C), with an increase after 7:00 p.m., starting with the reduction in temperature.

**Figure 4:**
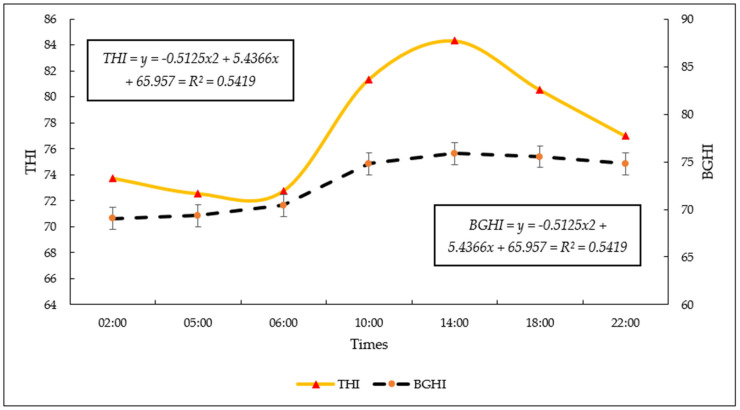
Temperature and Humidity Index (THI) and Black Globe Humidity Index (BGHI), at different times of the day, in the Eastern Amazon.

In relation to RR, there were significant interactions (*p* = 0.0214) between systems and schedules; in general, higher RR results were obtained in the traditional system. The RT of the animals did not differ between production systems (Table 2); however, there were significant statistical differences in relation to the collection time (*p* < 0.0001), with a quadratic effect (Figure 4), with the equation represented as ŷ = 37.8425 + 0.1801x − 0.0051x2, with coefficient of determination (R2) equal to 0.89. The curve is shown in Figure 4. BST showed no statistical difference between the regions studied or between the SP (35.6 °C) and TS (36.25 °C) systems.

At the THI, it was possible to observe that there was mild stress in the period from 10:00 p.m. to 6:00 a.m. and moderate stress in the period of greatest temperature rise, from 10:00 a.m. to 6:00 p.m. It was observed that the animals were in a thermal comfort situation between 2:00 a.m. and 6:00 a.m. (Figure 4) and were in a warning situation between 10:00 a.m. and 10:00 p.m., therefore signaling thermal stress, this being the time of highest temperature and humidity percentage. In relation to the BGHI, it was noted that from 2:00 a.m. to 6:00 a.m. there was no thermal stress in the animals, but from 10:00 a.m. to 6:00 p.m. stress was noted, due to the danger zone being signaled.

RT in the TS showed a positive correlation with AT (r = 0.31507; *p* = 0.0477). RT in the SP showed a positive correlation with THI (r = 35583; *p* = 0.0242). On the other hand, RT in the SP (r = 0.42873; *p* = 0.0058) and ST (r = 0.51015; *p* = 0.0008) showed a positive correlation with BGHI. RR in the TS showed a positive correlation with BGHI (r = 0.44908; *p* = 0.0037) (Table 3).

With regard to RR, the standard error of the mean was 0.85 mpm and there was an interaction effect between time of day and production system, with significant differences between production systems at 6:00 a.m. and 12:00 p.m.; at both times, the TS system had the highest RR averages (Table 4).

**Figure 5:**
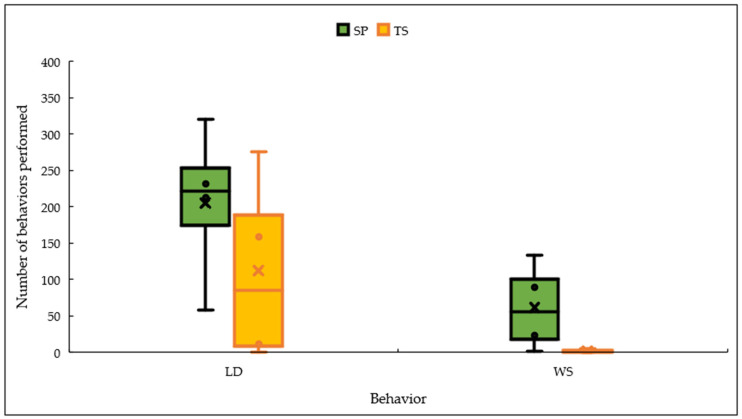
Rumination behavior while standing (WS) or lying down (LD) within the systems. TS = traditional system; SP = silvopastoral system.

The greatest amount of rumination was carried out by animals in the SP system, generally ruminating lying down (*p* < 0.05) (Figure 5).

**Figure 6:**
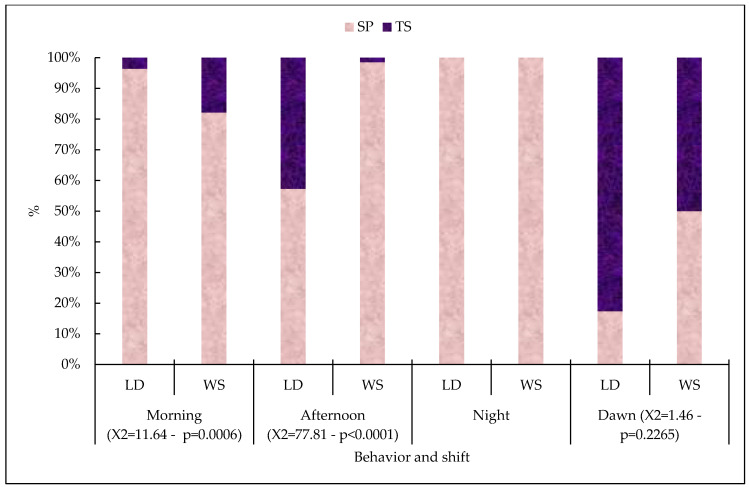
Relative frequencies (and percentages) for the behavior of animals ruminating lying down (LD) and standing up (WS), at different times and in each production system, with the associated chi-squared test (X2). LD = lying down ruminating; WS = ruminating while standing; TS = traditional system; SP = silvopastoral system.

With regard to rumination behavior in the morning and afternoon, there were higher numbers of WS and LD in the TS (*p* > 0.05). Most of the time, the cattle were LD during the morning and afternoon shifts, and at night and dawn they were WS in the TS (Figure 6).

**Figure 7:**
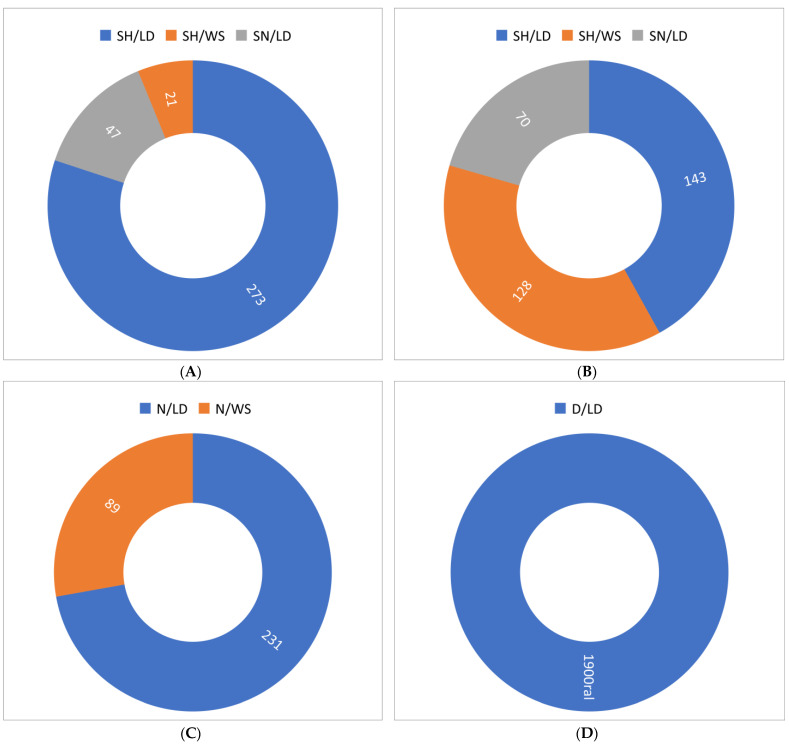
Behaviors according to day shift and production system. (**A**). Morning. (**B**). Afternoon. (**C**). Night. (**D**). Dawn. SH = shadow; SN = sun; N = night; D = dawn; LD = ruminating while lying down; WS = ruminating while standing.

The following behaviors are presented in reference to the rumination behaviors according to the shift of the day and the production system, with lower numbers of behaviors in the morning and early morning shifts (Figure 7A,D), and higher time fractions of this behavior in the afternoon and evening shifts (Figure 7B,C). Rumination is more abundant in the SP system.

## 4. Discussion

The variation observed in meteorological variables throughout the day, such as fluctuations in temperature and fluctuations in relative humidity, reflects the influences of the solar cycle and the typical climatic conditions of the region under study [17,30]. The increase in temperature during the period from 10:00 a.m. to 6:00 p.m. is consistent with the increase in direct solar radiation during the daylight hours, which culminates in its peak at 4:00 p.m. The high temperature observed at 16:00 (40 °C) is a reflection of the increase in solar radiation and heat accumulation throughout the day, and the relative humidity is higher during the night [18].

The fluctuation in relative humidity throughout the day can also be understood through the interactions between temperature and humidity. The lowest humidity values were seen at 20:00 in the evening, as more water droplets accumulated in the air, resulting in a drop in relative humidity. However, relative humidity tends to increase after 7 p.m. as the ambient temperature begins to drop, allowing the air to retain more moisture [18,39].

The significant difference in RR between the silvopastoral system and the traditional system can be understood considering the impacts of different environmental conditions, with the presence of shade in the silvopastoral system and management based on the respiratory behavior of cattle, as well as the absence of shade in the traditional system combined with solar radiation, promoting greater breathing intensity in an attempt to dissipate endogenous heat [50,51,52]. Direct exposure to sunlight can also increase the thermal load on animals, especially when shaded areas are not available, as evident in the traditional system [17,33,53,54].

The increase in RR in the traditional system may be associated with a series of factors. In conventional systems, in which cattle do not have access to adequate shade and are exposed to direct heat from the sun, thermal stress can be more pronounced. This can lead to an increase in the animals’ body temperature, causing them to seek to compensation for excess heat by increasing RR [18,55,56].

The reduction in RR observed in the silvopastoral system compared to the traditional system can be attributed to the more favorable thermal comfort conditions provided by the silvopastoral system. In this system, the presence of trees offers shade and protection against direct solar radiation, which helps to reduce heat stress in cattle. The milder ambient temperature provided by the shade of trees allows animals to maintain a more stable body temperature, reducing the need to increase RR to regulate temperature [16,57,58].

The RT showed differences (*p* < 0.05) between the times. This can be explained because animals placed in environments classified as high THI have difficulty dissipating heat, as they are exposed to temperatures beyond their tolerance, resulting in thermal stress. Under these conditions, endogenous heat production exceeds its cooling capacity, leading to a thermal shock that increases RT, resulting in indices above reference values [59,60,61,62,63,64].

The variation in THI throughout the day, as described, is related to the oscillations in temperature and relative humidity throughout the day. THI is a measure that combines air temperature and relative humidity to assess the potential heat stress to which animals are exposed. Higher THI values indicate a greater risk of heat stress [18].

The mild stress observed during the night and early morning (10:00 p.m. to 6:00 a.m.) can be explained by the combination of higher temperatures and relatively low relative humidity during these hours. Lower relative humidity contributes to a reduced ability of the air to cool animals through evaporation, increasing the risk of heat stress. Even though the air temperature is milder at night, low relative humidity can make the environment less favorable for heat dissipation by animals [2].

The moderate thermal stress observed during the period of rising temperatures (9:30 a.m. to 7:00 p.m.) is related to the increase in air temperature and the interaction with relative humidity. As the temperature rises, relative humidity tends to decrease, increasing the cooling demand for animals. This increase in temperature and reduction in relative humidity can result in an environment in which cattle have greater difficulty regulating their body temperature, leading to heat stress [18]. In addition, during this period, direct exposure to sunlight can result in excessive heat accumulation in animals, causing an increase in body temperature and making their thermoregulation capacity more challenging [39,65,66].

The variation in thermal comfort and thermal stress levels throughout the day, as described, is closely related to climatic conditions and the animals’ ability to adapt to fluctuations in temperatures and humidity. The thermal comfort of animals is determined by the balance between body temperature and environmental conditions, while thermal stress occurs when this balance is impaired, compromising the animals’ well-being [67,68,69].

The period of thermal comfort observed during the dawn and early morning [02:00 to 06:00 in the morning] is characterized by milder temperatures and relatively high humidity. These conditions are suitable for cattle to maintain their body temperature within acceptable limits, since the ambient temperature is not excessively high and humidity favors the evaporation of moisture through the respiratory tract, helping with cooling [65].

On the other hand, the period of thermal stress identified between 10:00 a.m. in the morning and 10 p.m. at night is attributed to the increase in temperature combined with the higher percentage of humidity. Conditions with high temperature and higher humidity can limit cattle’s ability to dissipate heat efficiently, leading to heat stress. The lack of effective cooling mechanisms, such as profuse sweating, makes cattle particularly susceptible to these conditions [70,71,72].

As environmental temperatures increase, cattle, need to implement physiological mechanisms to mitigate the increase in body temperature [73]. One of these mechanisms is the increase in RR, which promotes heat evaporation through the increased exchange of breathed air. This leads to a reduction in the body’s internal temperature, as evidenced by RT [74,75,76].

The THI and BGHI are measurements that combine AT with RH, providing a more comprehensive assessment of thermal conditions. When THI increases, it indicates an environment where air temperature is high relative to humidity, which can contribute to greater heat stress in animals [39].

The higher RR and RT in the TS can be attributed to various factors related to environment and animal management. Firstly, the absence of trees makes temperatures higher in the environment, especially during periods of intense heat. The increase in AT can lead animals to tachypnea as a mechanism for dissipating excessive heat, resulting in a higher RR [77,78].

In this same scenario, due to the lack of shade, heat stress is observed in animals, especially in regions with hot climates. Heat stress can cause a variety of physiological responses, such as a failure in thermoregulation or an increase in RR, which can be more pronounced in TS, where preferences for cooling or shelter can be limited, a fact observed in this study and confirmed by the THI and BGHI indices [39,79].

The effects of the interaction between time of day and rearing system play different roles in determining RR. The significant variations in average RR between the rearing systems at 6:00 a.m. and 12:00 p.m. indicated a dynamic response by the animals to the environmental and management circumstances throughout the day [80,81]. The higher RR and RT averages evaluated in the TS system at similar times represent a specific adaptation of the systems to metabolic needs or to the environment during these periods. In this way, the lack of varied management strategies based on the time of day and the rearing system can be studied in order to improve animal welfare and quality, leading to more effective practices in animal production [82,83].

Rumination, whether lying down or standing up, was affected by the time of day and the systems. The LD behavior observed most of the time in the SP animals can be explained by the relationship with their management and the environment in which they were being raised, since the availability of shade and shelter during the intense heat of the day provides more comfort for rest periods, leaving them more relaxed to perform their natural behaviors [84,85].

Rearing cattle in shaded environments offers stimuli due to the presence of trees, which also favors rumination, providing quieter areas that reduce animal stress caused by adverse climatic variables [86]. In addition, the quality and availability of forage are higher in the SP, which also influences rumination behavior, causing the animals to spend most of their time ruminating, contributing to better digestion and rest, impacting well-being and consequently animal productivity [87,88].

Thus, it is worth noting that in the SP during the morning and afternoon periods the animals remained LD, and during the night and early morning periods the ST animals had a preference for WS behavior, thus suggesting an adaptation to periods of activity and rest. These behavioral patterns can be explained by various factors such as environmental conditions, management in each system and the complexities and interactions between the environment and animal behavior [89,90,91].

## 5. Conclusions

The traditional system showed differences between the systems and the times of day, with the highest RR, signaling an attempt by the animals to adapt more physiologically in this system, which was not noticeable in the RT for the systems, but between the times of day. In addition, the THI and BGHI indices indicated a comfort zone in the early morning, specifically between 2:00 a.m. and 6:00 a.m., and stress between 10:00 a.m. and 6:00 p.m. The increase in RR and RT is related to the increase in AT. Associated with this, it was noted that the animals’ LD rumination took place to a greater extent in the SP, where there was shade. During the shifts with the highest thermal radiation, i.e., morning and afternoon, the animals tried to ruminate either in LD or WS. At night or in the early hours of the morning, the cattle tried to ruminate in the WS, especially in the TS.

Therefore, the SP system offers advantages for the thermal comfort of the cattle, with better RR and RT indices and a lower stress index compared to the TS system, which is confirmed by the higher rumination index in the SP system.

It is recommended that producers adopt measures to mitigate heat stress in the TS. For the TS, where RR values were highest, it is advisable to provide adequate shading and ensure good ventilation conditions and water availability. In addition, access to cooler areas during periods of rising temperatures can help minimize the impact of heat. In addition, it is recommended to adopt targeted measures such as adjustments to reproductive management periods and investments in infrastructure to promote the thermal comfort of animals in both breeding practices, favoring productive and reproductive performance.

## Figures and Tables

**Table 1:** Chemical composition of forage and components present in the concentrate fed to male Nelore cattle managed in different production systems in the Eastern Amazon.

Items (%)	System		
	Traditional	Silvopastoral	Silage *
DM	32.33	34.17	29.21
OM	91.96	91.96	91.88
MM	8.04	8.04	8.12
CP	7.69	6.47	4.22
EE	1.81	2.03	1.05
NDF	68.06	69.95	82.67
ADF	96.63	120.39	90.80
Components		Quantities per 100 kg	
Ground sorghum		72	
Soybean meal		5	
Urea		1	
White salt		1	
Evo core cut		5	
Cotton pie		16	
Total, Kg		100	

Notes: Diets (n = 2 systems) fed to Nelore cattle raised in three types of production systems. DM, dry matter; OM, organic matter; MM, mineral matter; CP, crude protein; EE, ether extract; NDF, neutral detergent fiber; ADF, acid detergent fiber. * This silage was supplied to all the systems.

**Table 2:** Breakdown of time depending on the production system.

Times	System	RT ^(1)^	RR ^(1)^
		Average	Average
6:00 ^(1)^	SP	38.80 ± 0.41	26.80 ± 3.91 a
6:00 ^(1)^	TS	38.76 ± 0.29	38.50 ± 7.10 b
12:00 ^(1)^	SP	39.11 ± 0.30	27.60 ± 2.17 a
12:00 ^(1)^	TS	39.2 ± 0.19	40.50 ± 6.31 b
18:00 ^(1)^	SP	39.57 ± 0.35	32.80 ± 3.42 a
18:00 ^(1)^	TS	39.55 ± 0.29	39.60 ± 5.05 b
0:00 ^(1)^	SP	39.29 ± 0.38	30.80 ± 3.79 a
0:00 ^(1)^	TS	39.12 ± 0.32	35.80 ± 2.39 b
Standard Error		0.1036	1.0241

Note: RT = rectal temperature; RR = respiratory rate; SD = standard deviation. Different letters (a, b) in the line indicate statistical differences (*p*  <  0.05). ^(1)^ There was a statistical difference between the times of day (*p* < 0.05). SP = silvopastoral; TS = traditional.

**Table 3:** Positive and negative correlations between the variables AT, RH, THI and BGHI were also observed in all shifts according to the experimental treatment.

**Variables**	**Strength of r and** ***p*-Value**	**AT**	**RH**
RT—SP	r	0.31507	−0.25681
	*p*-value	0.0477	0.1097
RT—TS	r	0.19143	−0.10024
	*p*-value	0.2367	0.5383
RR—SP	r	0.08825	−0.14135
	*p*-value	0.5882	0.3843
RR—TS	r	0.05727	0.03596
	*p*-value	0.7256	0.8257
**Variables**	**Strength of r and ** ***p*-Value**	**Comfort Index**	
		**THI**	**BGHI**
RT—SP	r	0.35583	0.42873
	*p*-value	0.0242	0.0058
RT—TS	r	0.26759	0.51015
	*p*-value	0.0951	0.0008
RR—SP	r	0.05978	−0.06537
	*p*-value	0.7140	0.6886
RR—TS	r	0.14128	0.44908
	*p*-value	0.3845	0.0037

Note: AT = air temperature; RH = relative humidity; THI = Temperature and Humidity Index; BGHI = Black Globe Humidity Index; RT = rectal temperature; RR = respiratory rate; BST = body surface temperature. SP = silvopastoral system; TS = traditional system. The correlation was considered positive or negative when the *p*-value was less than 0.05.

**Table 4:** Average and standard deviations (SD) for RR at different times and in each production system.

Times	SP	TS
	Average	Average
00:00	31.2 ± 7.25 a	32.4 ± 6.09 a
06:00	26.4 ± 6.58 b ^(1)^	41.4± 9.61 a
12:00	25.6 ± 2.06 b	39.8 ± 10.08 a
18:00	35.6 ± 6.65 a	39.6± 8.09 a

^(1)^ Averages followed by different letters (a, b) in the row differ by the F test, with a significance level of 0.05. TS = traditional system; SP = silvopastoral system. SD = standard deviation.

## Data Availability

The data presented in this study are available upon reasonable request from the corresponding author.
